# The Effect of Trabeculectomy Approach on Surgically Induced Astigmatism in the Treatment of Open-Angle Glaucoma

**DOI:** 10.7759/cureus.97955

**Published:** 2025-11-27

**Authors:** Hend M Al-Kaylani, Hansen Dang, Sean S Rivera, Christian Mays, Erin Boese, Nathaniel C Sears, Andrew E Pouw

**Affiliations:** 1 Ophthalmology and Visual Sciences, The University of Iowa Roy J. and Lucille A. Carver College of Medicine, Iowa City, USA; 2 Ophthalmology, University of California Los Angeles, Los Angeles, USA; 3 Ophthalmology, Eye Associates of New Mexico, Albuquerque, USA; 4 Ophthalmology, Eye Physicians and Surgeons of Augusta, PC, Augusta, USA; 5 Ophthalmology and Visual Sciences, The University of Iowa Hospitals and Clinics, Iowa City, USA; 6 Ophthalmology, Coastal Surgical Center, Newington, USA

**Keywords:** fornix-based trabeculectomy, glaucoma, limbus-based trabeculectomy, surgically induced astigmatism, trabeculectomy

## Abstract

Purpose

In open-angle glaucoma, trabeculectomy effectively reduces intraocular pressure when medical or laser therapies fail; yet, it typically introduces astigmatism. We conducted a retrospective observational study comparing surgically induced astigmatism (SIA) across three trabeculectomy methods (limbal-based and fornix-based with or without partial-thickness limbal corneal incision) by three surgeons at the University of Iowa Health Care.

Methods

A chart review was conducted for patients who underwent trabeculectomy for open-angle glaucoma at the University of Iowa Health Care between January 2018 and August 2023. Exclusion criteria included combined phacotrabeculectomy, retinal/corneal comorbidities, concurrent ocular surgeries, and patients under 18 years of age. Pre- and post-operative refractions within two years of surgery were used to calculate SIA. One-tailed analysis of variance (ANOVA), Tukey’s Honest Significant Difference test, and chi-square test of independence were performed to compare the surgical approach on SIA and change in the axis of astigmatism.

Results

The final sample included 87 patients with a mean age of 73.1±11.7 years; 59.8% of patients were women. Twenty-five procedures had a limbus-based approach by Surgeon 1, 23 fornix-based by Surgeon 1, 18 fornix-based by Surgeon 2, and 21 fornix-based by Surgeon 3. While mean SIA ranged from +0.913±0.617 diopters to +1.35±1.47 diopters, there was no significant difference between surgical approaches (p=0.409). Change in axis (p=0.451) or direction of change towards with-the-rule or against-the-rule (p=0.144) did not differ between the surgical approaches.

Conclusion

This study compared SIA after three trabeculectomy approaches in a single institution. Within our sample, surgical approach was not associated with SIA.

## Introduction

Open-angle glaucoma is a chronic disease that decreases the patient's quality of life through progressive, irreversible vision loss [1]. Most treatments aim to slow the loss of retinal ganglion cells by way of intraocular pressure (IOP) control [1,2]. If medical or laser therapies fail to control IOP or when vision loss is severe at initial presentation, trabeculectomy is an incisional procedure that can be pursued to control IOP [3]. Trabeculectomy has historically been a highly effective option for surgical treatment of open-angle glaucoma, and while new minimally invasive surgical options have been since been introduced, trabeculectomy will keep its role among surgical options, especially for cases of severe glaucoma [4].

Trabeculectomy is the ab externo creation of an ostium covered by partial-thickness scleral flap to increase or restore outflow from the anterior chamber into the subconjunctival space [5]. The approach through the conjunctiva to access bare sclera can either be limbus-based (in which the primary conjunctival incision is made posterior to the limbus) or fornix-based (peritomy-based incision made at the limbus). Fornix- and limbus-based trabeculectomy approaches have been directly compared in terms of bleb failure rates, visual acuity, and risk of infection [6-8]. A fornix-based approach is associated with greater success in creating a diffuse bleb with a normal vascular pattern [9] and, in one study, had lower rates of posterior capsule rupture during cataract extraction combined with trabeculectomy [6]. A 2021 review found that post-surgical IOP, number of anti-glaucoma medications at 24 months post-procedure, and frequency of adverse events were comparable between the two techniques. They also noted uncertainty in their conclusions due to limited research on the topic [10]. A comparative study conducted at a single institution found that late bleb-associated infections were more frequent with a limbus-based approach, while a fornix-based approach was associated with a higher risk of post-procedure hypotony as well as earlier cataract development requiring surgery [11]. Further, surgeon preference may play a role when choosing the surgical approach.

Some trabeculectomy surgeons additionally utilize a conjunctival closure technique to improve patient postoperative comfort and reduce bleb leak incidence via central mattress sutures anchoring the central conjunctival peritomy to a partial-thickness grooved incision in the peripheral cornea, in which the suture knot can be buried [12]. While the effect of multiple corneal grooves for closure on SIA has been studied [13], a literature search evaluating SIA from a single central corneal groove closure incision does not return any results. By altering the shape of the peripheral corneal skirt via bleb formation, trabeculectomy induces astigmatism [14]. SIA can negatively impact patient outcomes by creating glare, halos, and reduced visual acuity [15].

This retrospective cohort study compares the effect of trabeculectomy method on SIA. Limbus-based and fornix-based trabeculectomy utilizing a single central corneal groove incision for closure by three surgeons at the University of Iowa Hospitals and Clinics (AP, EB, NS) are compared.

## Materials and methods

All trabeculectomy procedures in this retrospective cohort study were completed at the University of Iowa Health Care by one of three surgeons (AP, EB, NS) between January 2018 and August 2023. These procedures were collected by querying the electronic medical records for all trabeculectomy procedures identified via CPT procedural code, done between January 2018 and August 2023. This study was approved by the Institutional Review Board at the University of Iowa. This research adhered to the tenets of the Declaration of Helsinki.

**Figure 1:**
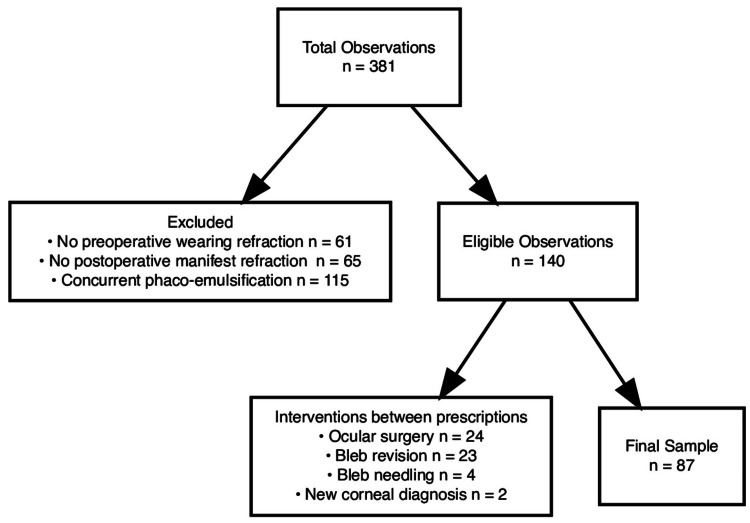
Consort diagram of exclusion criteria applied to arrive at our final sample of 87 procedures.

The initial sample included 381 surgeries. Exclusion criteria included absence of pre- or post-operative refraction data within two years before the date of surgery, absence of IOP recording within two months before surgery, or absence of IOP recording within five to eight weeks after surgery. Additionally, exclusion criteria included other ocular surgery done in the same eye in the timeframe between the collected pre- and post-operative refractions. Comorbidities diagnosed in the timeframe between the collected pre- and post-operative refractions, including corneal dystrophies, keratitis, keratoconus, hypotony, or corneal changes related to abnormal IOP were also excluded. Only eyes with the absence of any pre- and post-operative corneal pathologies were included. The final sample consisted of 87 surgeries (Figure 1).

The pre- and post-operative refractions noted within two years of the procedure were compared via subtraction to evaluate for SIA change. One-tailed analysis of variance (ANOVA) and Tukey’s Honest Significant Difference (HSD) test were performed to compare induced astigmatism and change in axis (degrees). A chi-square test of independence was used to compare the change in the direction of astigmatism between surgical approaches (toward with-the-rule, toward against-the-rule, or no change; no change defined as ≤5 degrees).

Surgical technique

Surgeon 1

Patients received a drop of topical tetracaine followed by an injection of 0.1 mL of 0.1 mg/mL mitomycin c mixed with 0.25% bupivacaine with 1:200,000 epinephrine into the superior subconjunctival space. The ocular surface was then copiously irrigated with a balanced salt solution. A retrobulbar block was given and the eye prepped and draped. A 7-0 polyglactin traction suture was placed through the superior cornea and the eye infraducted. For a fornix-based trabeculectomy, a conjunctival peritomy was performed at the limbus; for a limbus-based trabeculectomy, a conjunctival peritomy was performed 8 mm posterior to the limbus. Hemostasis was achieved with bipolar cautery. A partial thickness scleral flap was made posterior to the limbus and carried forward to the limbus. A temporal paracentesis was made, and the anterior chamber infused with a miotic agent. The anterior chamber was then entered under the scleral flap, and the sclerostomy enlarged with a Kelly punch. A peripheral iridectomy was performed. The scleral flap was closed with 10-0 nylon sutures. The conjunctival peritomy was closed with two 8-0 polyglactin wing sutures for the fornix-based trabeculectomy and a running 8-0 polyglactin suture for the limbus-based trabeculectomy. The eye was filled with balanced salt solution and a watertight bleb confirmed. Subconjunctival antibiotic and steroid were given. Patients received a drop of moxifloxacin, prednisolone, atropine, and neomycin-polymyxin-dexamethasone ointment and a patch and shield. 

Surgeon 2 

Patients were given a retrobulbar block and the eye prepped and draped. A 7-0 polyglactin traction suture was placed through the superior cornea and the eye infraducted. A conjunctival peritomy was performed at the limbus to create a fornix-based trabeculectomy. Hemostasis was achieved with bipolar cautery. Mitomycin c (0.2 mg/mL to 0.4 mg/mL) soaked sponges were placed in the subconjunctival space and allowed to sit for 60 seconds. The sponges were then removed and the ocular surface copiously irrigated with a balanced salt solution. A partial thickness scleral flap was made posterior to the limbus and carried forward to the limbus. A temporal paracentesis was made and the anterior chamber infused with a miotic agent. The anterior chamber was then entered under the scleral flap, and the sclerostomy enlarged with a Kelly punch. A peripheral iridectomy was performed. The scleral flap was then closed with 10-0 nylon sutures. The conjunctival peritomy was then closed with two 10-0 polyglactin wing sutures. The eye was then filled with a balanced salt solution and a watertight bleb observed. Subconjunctival antibiotic and steroid were given. Patients then received a drop of moxifloxacin, prednisolone, atropine, and neomycin-polymyxin-dexamethasone ointment and a patch and shield.

Surgeon 3

Patients were given a drop of topical tetracaine and in some rare cases a retrobulbar block. A 7-0 polyglactin traction suture was then placed through the superior cornea and the eye infraducted. A small conjunctival snip incision was made at the limbus and 0.2 ml of 2% lidocaine with 1:10,000 epinephrine, followed by 0.2 ml of mitomycin C (0.4 mg/mL), were injected into the subconjunctival space. The ocular surface was then copiously irrigated with a balanced salt solution. The conjunctival snip incision was then enlarged along the limbus to create a fornix-based limbal peritomy. Hemostasis was achieved with bipolar cautery. A partial thickness scleral flap was made posterior to the limbus and carried forward to the limbus. A temporal paracentesis was made and a partial thickness central corneal groove incised into the superior peripheral cornea near the limbus. The anterior chamber was then entered under the scleral flap, and the sclerostomy enlarged with a Kelly punch. A peripheral iridectomy was performed. The scleral flap was then closed with 10-0 nylon sutures. The conjunctival peritomy was then closed with two 10-0 polyglactin wing sutures and a central 10-0 polyglactin mattress suture with the knot buried into the partial thickness central corneal groove. The eye was then filled with a balanced salt solution and a watertight bleb confirmed. Patients then received a drop of moxifloxacin, prednisolone, and neomycin-polymyxin-dexamethasone ointment and the eye shielded (the eye was additionally bandage patched if a retrobulbar block had been administered).

For all retrobulbar blocks, 5 cc of 1% lidocaine, 0.37% bupivacaine and 37 units of hyaluronidase were used. For all surgeons, the postoperative intraocular pressure was titrated by adjusting topical steroid regimen, digital ocular compression, and laser suture lysis as needed.

## Results

The final sample consisted of 87 procedures; exclusion criteria were applied as shown in Figure 1. Of the 381 initial procedures, 115 (30.2%) included concurrent phacoemulsification, which were excluded from the study due to the confounding effect of astigmatism changes inherent in cataract removal. Sample demographic information is presented in Table 1.

**Table 1:** Demographics of our final sample.

	Total (N=87)	Surgeon 1, Limbus (N=25)	Surgeon 1, Fornix (N=23)	Surgeon 2, Fornix (N=18)	Surgeon 3, Fornix with CCG* (N=21)
Age (years)					
Mean (SD)	73.1 (11.7)	73.2 (11.5)	70.3 (15.7)	75.6 (6.03)	74.1 (10.6)
Median (Min, Max)	74.0 (42.0, 90.0)	74.0 (50.0, 90.0)	67.0 (42.0, 88.0)	73.5 (66.0, 84.0)	76.0 (51.0, 89.0)
Sex					
Female	52 (59.8%)	11 (44.0%)	17 (73.9%)	13 (72.2%)	11 (52.4%)
Male	35 (40.2%)	14 (56.0%)	6 (26.1%)	5 (27.8%)	10 (47.6%)
Pre-operative Cylinder (diopters)					
Mean (SD)	1.03 (0.967)	0.940 (0.885)	0.913 (0.677)	0.944 (0.572)	1.35 (1.47)
Median (Min, Max)	0.750 (-1.00, 6.25)	0.750 (-1.00, 2.50)	0.750 (0, 2.50)	1.13 (0, 2.00)	0.750 (0, 6.25)
Post-operative Cylinder					
Mean (SD)	1.18 (0.931)	1.20 (0.760)	0.946 (0.617)	1.07 (0.658)	1.50 (1.44)
Median (Min, Max)	1.00 (0, 5.57)	1.00 (0, 2.75)	1.00 (0, 2.50)	1.00 (0, 2.25)	1.00 (0, 5.57)
Cylinder Difference					
Mean (SD)	0.198 (0.691)	0.280 (0.814)	0.0326 (0.507)	0.319 (0.756)	0.179 (0.657)
Median (Min, Max)	0.250 (-1.00, 2.75)	0.250 (-0.750, 2.75)	0 (-1.00, 1.25)	0.250 (-0.750, 2.25)	0.250 (-1.00, 1.25)

**Figure 2:**
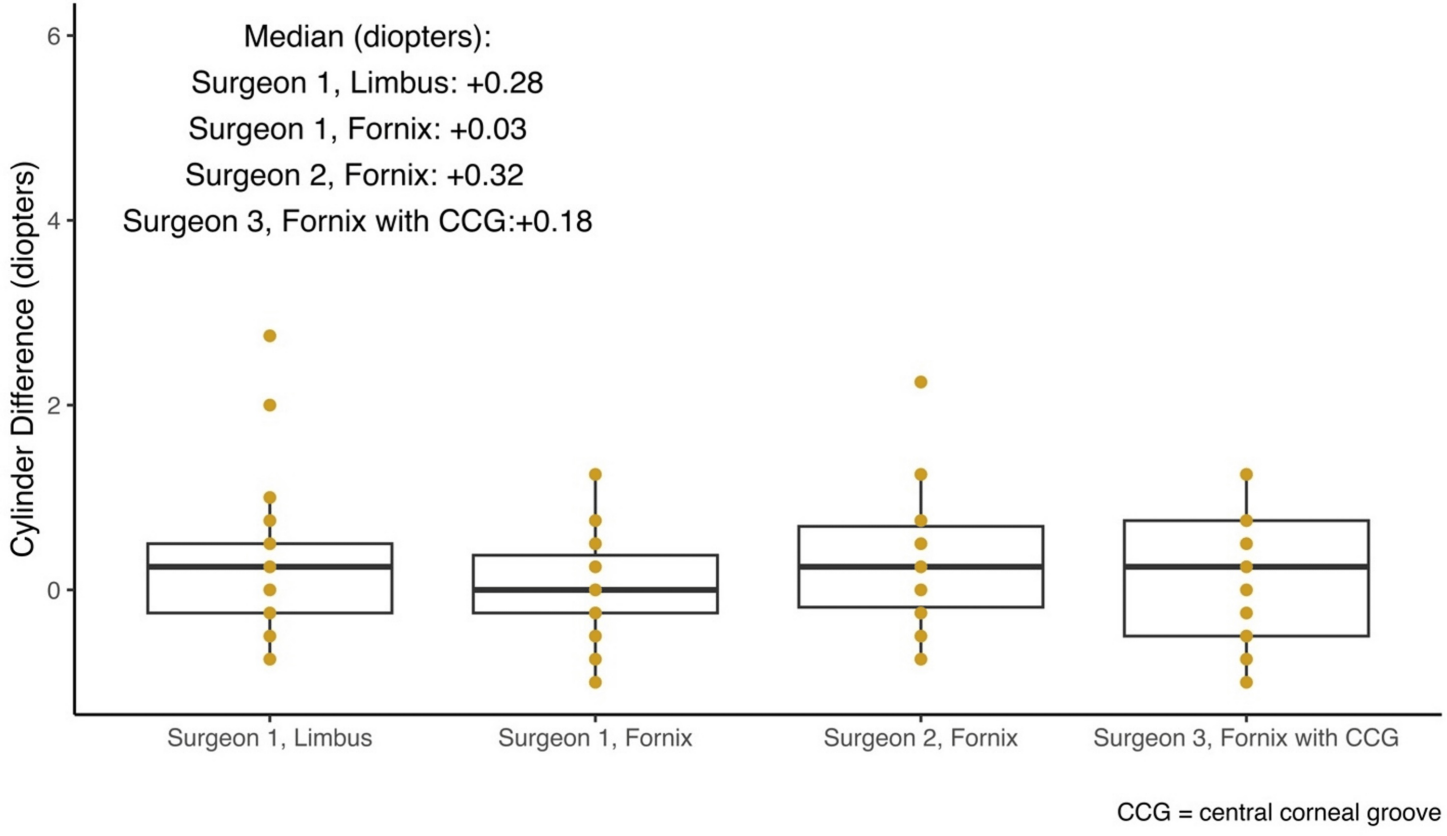
Median pre- and post-operative cylinder measurements compared between trabeculectomy methods.

The primary outcome of the study was to determine the postoperative change in astigmatism. All surgical approaches studied increased the magnitude of astigmatism in the patient’s postoperative manifest refraction (Figure 2). 

A one-tailed ANOVA demonstrated that mean induced astigmatism did not statistically differ between surgical approaches (F(3,83)=(0.974), p=0.409). Tukey’s HSD test for multiple comparisons found that the mean induced astigmatism did not significantly differ between any of the four groups.

As presented in Table 2, the axis of astigmatism changed by an average of 19 degrees across the full sample and did not differ between surgical approach (F(3,83)=(0.888), p=0.451). Surgical approach was not associated with the direction of change toward with-the-rule or toward against-the-rule astigmatism (p=0.145, df=6, χ^2^=9.579).

**Table 2:** Astigmatism characteristics of our sample. CCG: Central corneal groove.

	Total (N=87)	Surgeon 1, Limbus (N=25)	Surgeon 1, Fornix (N=23)	Surgeon 2, Fornix (N=18)	Surgeon 3, Fornix with CCG (N=21)
Change in cylinder					
Mean (SD)	0.198 (0.691)	0.280 (0.814)	0.0326 (0.507)	0.319 (0.756)	0.179 (0.657)
Median [Min, Max]	0.250 (-1.00, 2.75)	0.250 (-0.750, 2.75)	0 (-1.00, 1.25)	0.250 (-0.750, 2.25)	0.250 (-1.00, 1.25)
Axis Change (degrees)					
Mean (SD)	19.0 (19.3)	14.4 (15.3)	19.0 (21.6)	19.8 (23.0)	23.7 (17.7)
Median (Min, Max)	14.0 (0, 86.0)	10.0 (0, 71.0)	15.0 (0, 86.0)	10.0 (0, 71.0)	17.0 (2.00, 69.0)
Direction of Axis Change					
No change	23 (26.4%)	8 (32.0%)	8 (34.8%)	6 (33.3%)	1 (4.8%)
Toward against the rule	34 (39.1%)	9 (36.0%)	9 (39.1%)	8 (44.4%)	8 (38.1%)
Toward with the rule	30 (34.5%)	8 (32.0%)	6 (26.1%)	4 (22.2%)	12 (57.1%)

## Discussion

The goal of this retrospective observational study was to compare the effect of trabeculectomy method (limbus-based, fornix-based with or without limbal relaxed incision) on SIA. Within our sample, we found that all the methods increase astigmatism by approximately one diopter and that the differences between each method were not statistically significant. We also found that the change in axis and direction of change toward with-the-rule or against-the-rule astigmatism does not differ between surgical approaches. Previous work on the effect of trabeculectomy on SIA similarly found a median increase in astigmatism (+0.5 D in their sample), with most cases not requiring new glasses or corrective wear [16]. Previous work does not compare the effect of the trabeculectomy method (limbus- versus fornix-based) on SIA.

While there is evidence comparing limbus- and fornix-based trabeculectomy on the failure rate, change in pressure, and number of medications required post-operatively, there is limited work comparing change in astigmatism [10,11,17]. Thus, this study adds insight into the effect of the trabeculectomy approach on SIA. The more elderly mean age of our sample (73.1±11.7 years of age at time of surgery) improves the reliability of our findings, because the sclera is more rigid and therefore scleral flexibility is less of a confounding variable on SIA [18]. Future work comparing SIA of these approaches can guide clinicians in predicting refractive changes, individualizing treatment decisions, and advising patients on what to expect from their vision after surgery. Future work could also investigate the number of post-operative visits, on average, initiated by patients to optimize refractive measurements.

The limitations of this study are that some post-surgery measurements were taken less than one year post-operatively and surgeon techniques differed with the number of sutures used to secure the scleral flap and degree of cautery in addition to limbal or fornix-based approaches. Studies on astigmatism measured one year post-operatively vary [14,19]. A review by Pakravan et al. in 2017 found that a mean of +0.7 D of SIA remains at 12 months post-operatively [20].

It is important to note that our analysis utilizes post-operative refraction as a metric of astigmatism rather than topography data, an unbiased objective measurement, and has been used in multiple studies of post-surgical astigmatism [21-23]. While refraction offers a less accurate measurement of astigmatism, it is an easily accessible measurement and is adequate for patient-centered needs such detecting the need for new glasses post-operatively. Topographic data was not available for this retrospective study, as it was not routinely obtained in our clinic’s typical glaucoma practice.

Due to the necessity of excluding many trabeculectomy surgeries to meet exclusion criteria, our study is unfortunately underpowered (see Supplemental Text: Power Analysis). However, we hope that our results that are suggestive of no significant differences in surgical-induced astigmatism between the studied trabeculectomy techniques are of value in directing further investigations of these techniques and their effects on astigmatic outcomes. 

Further, manifest refraction measurement of astigmatism in our study does not consider corneal topography or axial eye length [21]. Additionally, lack of central vision and diminished contrast sensitivity in advanced glaucoma cohorts may limit or mask the true measurement of astigmatism with manifest refraction. Topographic changes can last up to 12 months and can fluctuate [14]. From a clinical standpoint, measurement of refractive astigmatism is sufficient when considering post-surgical quality of life for a patient.

## Conclusions

While trabeculectomy does induce astigmatism, fornix- and limbus-based techniques do not significantly differ in the strength, axis, or direction of astigmatism. The retrospective nature of our study is a limitation. Future work in a prospective setting can ensure that all post-operative measurements are collected at a similar timeframe, increasing the accuracy of all comparisons. A prospective study design would also be able to include corneal topographic data.

## Appendices

Supplemental text: power analysis

An a priori power analysis was conducted using G*Power version 3.1.9.7 [24] for sample size estimation, based on pilot data previously collected in our clinic, which compared the average trabeculectomy-induced cylinder for each surgeon. With a significance criterion of α=.05 and power=.80, the minimum sample size needed with this effect size is n=292 (n=73) per group for analysis of variance between four groups. We collected a total of 371 surgeries, representing years of clinical and surgical practice at a tertiary care institution. Out of this, 294 surgeries were excluded as per our exclusion criteria to mitigate confounding. The final sample size was 87.
